# Smartphone addiction and phubbing behavior among university students: A moderated mediation model by fear of missing out, social comparison, and loneliness

**DOI:** 10.3389/fpsyg.2022.1072551

**Published:** 2023-01-06

**Authors:** Ruqia Safdar Bajwa, Haslinda Abdullah, Zeinab Zaremohzzabieh, Wan Munira Wan Jaafar, Asnarulkhadi Abu Samah

**Affiliations:** ^1^Institute for Social Science Studies, Universiti Putra Malaysia, Serdang, Selangor, Malaysia; ^2^Faculty of Human Ecology, Universiti Putra Malaysia, Serdang, Selangor, Malaysia

**Keywords:** smartphone addiction, phubbing behavior, university students, fear of missing out, social comparison orientation, loneliness

## Abstract

**Introduction:**

This article examines mediators and moderators that may explain the link between smartphone addiction and phubbing behavior using a sample of 794 university students.

**Methods:**

A mediation model was tested to test the hypothesis that social comparison orientation and fear of missing out would mediate the link between smartphone addiction and phubbing behavior. Additionally, a moderated mediation model was leveraged to examine loneliness as a moderator within the hypothesized model. The data collected were analyzed using SPSS.

**Results and Discussion:**

The findings show a significant positive relationship between smartphone addiction and phubbing behavior. The findings confirm the hypothesized associations and reveal that smartphone addiction is positively linked to phubbing behavior. The link, on the other hand, is partially and sequentially mediated by the fear of missing out and social comparison orientation. As a result, both mediators might be regarded as proximal variables of phubbing behavior. Moreover, the associations between both smart addiction and phubbing behaviors as well as social comparison orientation and phubbing behaviors are moderated by loneliness. These two effects were stronger for university students with high loneliness than for those with low loneliness. This study addresses a major gap in the clinical psychology literature through the attempt to explore the relationship between smartphone addiction and increased phubbing behavior among university students.

## 1. Introduction

Recently, there exist numerous discussions among the higher education (HE) community about smartphones. Universities and colleges are examining more and more ways to utilize smartphones to increase student engagement and to better captivate them. As stated by Nand et al. (2020), smartphone use has a significant impact on how HE students act in social and academic contexts. Furthermore, HE students frequently use their smartphones for academic purposes (Nand et al., 2020). Researchers have recently expressed their concern about smartphones’ possible negative impacts on students’ psychological and physical health, as well as the quality of their social interactions, despite the devices’ clear benefits. The rise of “phubbing” (PHU), a new maladaptive behavior, is associated with smartphone usage’s increase in popularity. PHU is defined as a modern form of social snubbing, where a person ignores another in social interaction by focusing on their smartphones instead of engaging in a conversation (Nazir, 2017). This behavior embodies the actual negative effects of poor communication that harm interpersonal relationships and feelings of personal well-being.

Higher education students exhibit the effects of excessive smartphone usage in the form of attention deficit hyperactivity disorder and concentration difficulties. Nonetheless, the role of PHU in contributing to these symptoms is practically unknown. Within the Pakistani scenario, smartphone use disorder among HE students is surging, indicating the possibility of PHU among HE students (Bajwa et al., 2021). Furthermore, even in this day and age of e-learning, only a small percentage of students use their smartphones to supplement their learning. The vast majority of them use smartphones for personal communication, indicating that PHU may have many predictable or attendant factors. Recent studies have proposed that these phenomena may have some impacts on HE students’ academic outcomes, particularly those who engage in smartphone activities in class (e.g., Dontre, 2021). PHU has been found to cause psychological impairment or distress, loneliness, depressive states, insecurity, hopelessness, low self-esteem, somatization, and alexithymia (Fernández-Andújar et al., 2022). Such mental distresses may contribute to poor academic performance, personal relationships, social identity, and family relationships (Yang et al., 2019).

Against this backdrop, this present research aims to address the dearth of research on predictors of PHU behavior (PHUB) among HE students. This study’s focus is on Pakistan, where smartphone overuse among HE students has contributed to the upsurge in PHUB cases. A growing literature indicates that smartphone addiction (SMA) harms Pakistani HE students’ satisfaction with classroom connectedness (e.g., Tamura et al., 2017). Despite being a global phenomenon – not constrained within the context of Pakistan – very few studies have attempted to identify the predictive factors for PHUB in the HE setting. Based on past studies, some risk factors for PHUB include addictive behaviors (such as SMA), fear of missing out (FoMO), loneliness (Gong et al., 2019), and social comparison orientation (SCO; Chotpitayasunondh and Douglas, 2016; Blanca and Bendayan, 2018). Proper identification and acknowledgment of these factors might be essential for ensuring that the right preventive measures and treatments are implemented to decrease pathological PHUBs and problematic smartphone usage. Furthermore, in most studies on smartphone use, addictive tendencies – most importantly, smartphone addiction – is among the top predictors of PHUBs (e.g., Błachnio and Przepiorka, 2019; Balta et al., 2020). The pathological use of smartphones – at the price of worsening relational connections brought about by PHUBs – has also been highlighted by Salehan and Negahban (2013) in the same vein. Recognizing the past findings in the literature, this current study highlights that SMA should be a strong predictor of PHUB.

FoMO and SCO have begun to be recognized as PHUB predictors by research on psychological aspects of PHU and SMA (Chotpitayasunondh and Douglas, 2016; Blanca and Bendayan, 2018; Franchina et al., 2018; Balta et al., 2020). On the other hand, loneliness has been proven to exhibit a strong correlation with PHUB. In recent times, loneliness is becoming more prevalent among the younger generation. According to Karadağ et al. (2015) HE students prefer to use their smartphones more often when they feel lonely, as they believe that doing so will mitigate their loneliness. Additionally, research has shown that high FoMO will promote problematic smartphone use, further increasing the risk of PHU. However, there has not exist much empirical study on the relationship between SMA and PHUB, particularly in the Pakistani context (Chotpitayasunondh and Douglas, 2016; Butt and Arshad, 2021; Lai et al., 2022). Additionally, there is not much knowledge about the factors that might explain this relationship. Acknowledging this gap in the literature, the current study investigates how SMA and PHUB are directly and indirectly related.

## 2. Theoretical background

This study employs the Social Comparison Theory (SCT; Festinger, 1964) and Compensatory Internet Usage Theory (TCIU; Kardefelt-Winther, 2014). TCIU depicts problematic usage as a coping or compensating mechanism for those who are going through destructive or deleterious psychological states (Wolniewicz et al., 2018). People with psychosocial issues may use their smartphones excessively to cope with life’s stressful situations. According to this theory, people’s excessive smartphone use may be a sign that they are experiencing distressful feelings like loneliness. Their interaction with smartphones serves to suppress these feelings (Elhai and Contractor, 2018). This fact might explain the connection between detrimental smartphone usage and negative emotional states like loneliness and FoMO (Chotpitayasunondh and Douglas, 2016; Dayapoglu et al., 2016; Bolkan and Griffin, 2017; Elhai et al., 2018), both of which are strongly associated with PHU (Chotpitayasunondh and Douglas, 2016). Nonetheless, some researchers feel that the TCIU presents a limited perspective and does not provide a thorough outlook on the investigation of the hypothesized correlations. This is even though TCIU can explain negative emotional states as a feasible trigger to predict HE students’ inclination for PHU. To complement this deficiency in the theory, the current study complements it with SCT to assess the associations between FoMO, social comparison (SC), SMA, loneliness, and PHU.

Additionally, SCT proposes that individuals may use upward or downward SCs, depending on their motives. The type of comparison that a person makes depends on their level of motivation (Talwar et al., 2019). For instance, a highly driven person might strive for personal development and perform upward SCs (Cramer et al., 2016). Prior research has revealed that users of social media (SM) sites may be prone to believing that other users are in comparatively better circumstances than they are. This will inevitably lead them to engage in upward SC (Latif et al., 2021). Inspired by the approach taken in past research, the current study uses SCT to investigate how SMA, FoMO, and PHU are related to SC in its upward version. Accordingly, in this study, individuals experiencing FoMO are also more prone to involve in upward SC, causing them to feel negative emotions like PHU. In acknowledgment of TCIU and SCT, this study constructs one moderator (loneliness) and two mediators (SCO and FoMO) to gain insights into the links between SMA and PHUB among Pakistani HE students.

### 2.1. Smartphone addiction and phubbing headings

According to several definitions, SMA is a behavioral addiction with four elements: compulsive smartphone usage, tolerance (defined as a person’s diminished sensitivity to an addictive chemical or stimuli as the result of repeated use), withdrawal, and functional impairment. Consequently, SMA is acknowledged as being equivalent to the Diagnostic and Statistical Manual of Mental Disorder criteria for internet addiction (DSM-V; Nuckols, 2013). Researchers have discovered that both PHUB and SMA harm people’s social functioning. According to earlier research, during the COVID-19 outbreak, HE students had to maintain their social distance, and may only gain information about the epidemic *via* smartphones. This has inevitably increased the amount of time and frequency spent on the device. As a result, many felt lonely and abandoned, making it harder for them to socialize with and build strong connections with others. When the movement restrictions placed on the students were eased – as the society transitioned into the endemic phase – undesirable behaviors such as phubbing began to surface.

Additionally, recent studies have shown that people will communicate with others online and use their smartphones more frequently to feel satisfied, raising the risks of SMA (Zhao et al., 2021; Diotaiuti et al., 2022). Lai et al. (2022), on the other hand, asserted that SMA is a predictor of PHUB in a sample of Chinese HE students. It was discovered *via* prior research that technology addictions, such as SMA, may predict PHUB (Al-Saggaf and O’Donnell, 2019). However, little study has been done so far on how SMA among Pakistani HE students relates to PHUB.

### 2.2. Fear of missing out and social comparison as mediators

SCO and FoMO can be taken into consideration in a study that examines the nature of phubbing, due to their theoretical connection with SMA and PHUB. According to SCT – a theory that explains how people get insight into who they are through comparison with others – SCO symbolizes an individual’s propensity to engage in SCs (Gibbons and Buunk, 1999). Additionally, in studying the relationship between SMA and PHUB, the psychological notion of FoMO – which may be viewed as a response variable of SC – may also be taken into account (Schmuck et al., 2019; He et al., 2020). FoMO is defined as “the need to always know what other people are up to” or “a continuous dread that one may be missing out on pleasant events while others are present” (Przybylski et al., 2013). Moreover, overusing smartphones can be linked to FoMO, which, in turn, may result in PHUBs (Hong et al., 2012).

A study has found that excessive smartphone usage and PHUB are both caused by FoMO in teenagers from Flemish communities while confirming the probable link between the two (Franchina et al., 2018). Later studies have also revealed that people with high levels of FoMO are more prone to abusing their smartphone usage and, as a result, indulge in PHUB fueled by their urge to be connected online (Balta et al., 2020). Additionally, certain empirical investigations suggest that SCO and FoMO may be linked to PHUB (Chotpitayasunondh and Douglas, 2016; Davey et al., 2018; Franchina et al., 2018; Balta et al., 2020). In a sample of 474 Italian adults, Servidio et al. (2021) revealed that the link between self-concept clarity and problematic smartphone use was explained by SCO and FoMO. To the best of our knowledge, no earlier research has studied how SCO and FoMO may have a mediation role in the connection between SMA and PHUB.

### 2.3. Loneliness as a moderator

Loneliness may be defined as the feeling a person gets when his/her social relationships are inadequate on either the qualitative or quantitative level (Peplau, 2022). According to an increasing number of studies, young individuals who are feeling lonely have a larger propensity to involve in internet-related addiction – such as problematic smartphone use (Liu et al., 2020). According to another meta-analysis, loneliness pushed people to use Internet-connected devices rather than vice versa (Song et al., 2014). Xu (2017), when studying the links between SMA, social anxiety, and loneliness among 195 high school students in Beijing, observed that people who use their phones excessively also suffer from increased loneliness. Various other studies have also looked at the mediation impact of loneliness in the linkages between other adverse family features – such as parental disagreement, technological indifference, and dysfunctional families – and Internet-related addiction behaviors (He et al., 2020). The link between PHUB and its determinants comprises one of the subjects of interest in this research. The decision to include this link within the current study is motivated by the fact that the function of loneliness as a moderator has received less attention in other studies.

### 2.4. The present study

**Figure 1:**
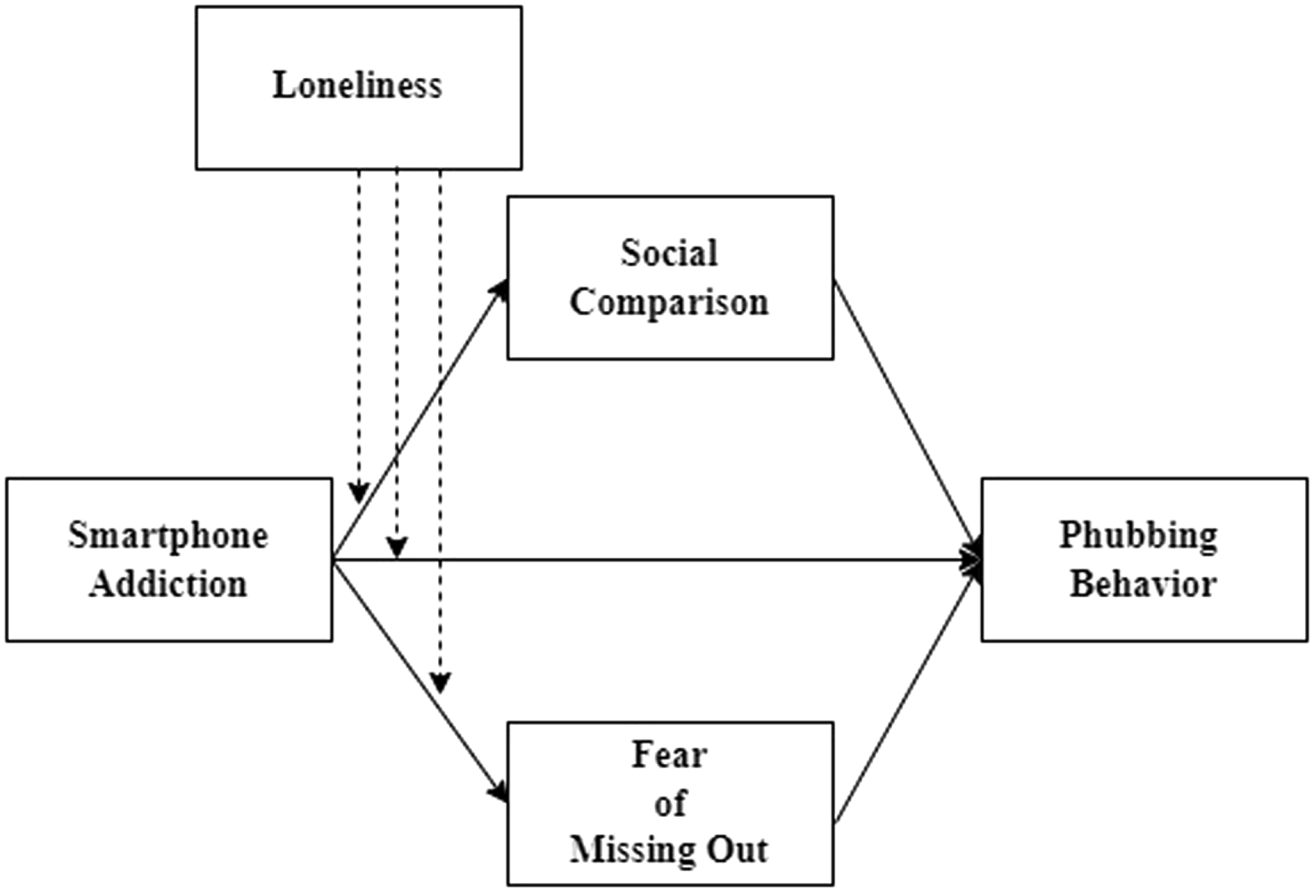
Research framework.

Prior research has mostly concentrated on the association between SMA and students’ PHUB scores. There are, however, very few studies that investigate the connection between SMA, loneliness, PHUB, SCO, and FoMO. It is unclear what mediating processes could explain this correlation and what moderating factors might have an impact on this relationship. In detail, the aims of the present study are several folds. Firstly, to explore if SCO and FoMO would affect HE students’ PHUB. Secondly, to determine if, in a serial mediation model, SCO and FoMO would moderate the relationship between SMA and PHUB among students. Thirdly, to determine if loneliness would act as a moderator in the direct and indirect relationships between SMA and PHUB *via* SCO and FoMO. Figure 1 depicts a segregated moderated mediation model whereby loneliness – *via* parallel pathways of SCO and FoMO – moderates the indirect effects of SMA on PHUB.

## 3. Materials and methods

### 3.1. Participants and procedures

A total of 800 university students participated in this study, and 794 usable questionnaires were returned (99.25% response rate). Participants were recruited *via* a multistage cluster random sampling technique. In South Punjab, Bahauddin Zakariya University and Islamia University Bahawalpur were randomly selected, and the faculties at each university were split into science and non-science groups. Three departments were selected randomly from each group, and two classes were selected from each department. 53% of the participants were female, aged 18–26, while 47% were male. All participants individually answered the questionnaires for around 20 min after giving informed consent to the researchers. The frequency of daily and weekly internet use was assessed. We found that 92% of the participants were identified as frequent users of the internet (almost every day). Additionally, 74% of participants prefer to use the internet from home.

### 3.2. Measures

The Smartphone Addiction Scale (Kwon et al., 2013) was employed to measure problematic smartphone use. It entails 10 items – such as “Missing scheduled work because of smartphone use” – measured using a 6-point Likert scale with 1 (never) to 6 (always). The higher average scores reflect teenage SMA. This scale has an excellent level of validity and reliability and is frequently used to evaluate problematic smartphone usage by Chinese youths (Wang et al., 2017). Furthermore, the Smartphone Addiction scale exhibits a Cronbach’s alpha of 0.91.

On top of that, the Phubbing Scale (Karadağ et al., 2015) was employed to asssess PHUB. This instrument contains 10 items – e.g., “I’m constantly on my phone while I’m with friends” – to which participants measure their response on a 5-point Likert scale ranging from 1 (never) to 5 (always). The scale has two dimensions: Communication Disturbance and Phone Obsession. In the original version of the measure, the internal consistency of the former subscale is 0.87, while the internal consistency of the latter is 0.85.

Moreover, the Social Comparison Scale was evaluated using an 11-item self-report scale derived from the INCOM (Gibbons and Buunk, 1999; Ruggieri et al., 2021). The scale measures the propensity for SC through the identification of key elements of the self, the other, and their psychological interactions. The items – e.g., “I usually prefer to know what others in a comparable circumstance would do” – are assessed on a five-point Likert scale ranging from 1 (I strongly disagree) to 5 (I strongly agree). Greater SC behaviors are indicated by higher scores. In the current investigation, Cronbach’s alpha was 0.78.

Adolescents’ loneliness, on the other hand, was measured using the 3-item Loneliness Scale (Hughes et al., 2004). This scale has been utilized with high reliability and validity in a previous study involving Chinese youths (Jiang et al., 2022). This scale consists of three items assessed on a 7-point scale ranging from 1 (never) to 7 (often) (always). The average scores for these items represent degrees of loneliness among adolescents, with a higher score indicating a greater sensation of loneliness. Cronbach’s alpha coefficient for this scale was 0.92 for the current study.

Besides that, the Fear of Missing Out scale established by Przybylski et al. (2013) was used to measure FoMO. It consists of 10 items – e.g., “It irritates me when I miss a chance to see friends” – that are rated on a 7-point Likert scale ranging from 1 (never) to 7 (always). This scale has been widely used in research involving adolescents with good reliability and validity (Ma et al., 2021). Higher average scores imply higher degrees of FoMO. Cronbach’s alpha coefficient for this scale was 0.92.

## 4. Data analysis

The researchers carried out data entry using a personal computer (PC) and analyzed the data using SPSS 26.0. The data were then evaluated in five phases using the procedures of multiple mediations and moderated mediation analyses described in previous studies (e.g., Hayes, 2017; Wang et al., 2017). The first phase involved a factor analysis to investigate common variances to check for common technique biases. The second phase then entailed descriptive and correlation analyses to examine the results of the five surveys. In the next phase, Model 6 and a multiple mediation model were used to assess the mechanisms of SCO and FoMO in the relationship between SMA and PHUB. Finally, the study examined whether loneliness moderated the direct and indirect effects of SMA on PHUB by exploring the moderated mediation model *via* Model 8 of the PROCESS macro v3.0.

## 5. Results

### 5.1. Common method biases test

In the current study, common method deviations were systematically reduced by utilizing partial item reverse measurements and anonymous assessment. However, the literature suggests that the outcomes of self-reported surveys may be biased by common methods (Kenny, 2021). To ascertain if bias exists, the current study applied Harman’s (1960) Single-Factor Test, as suggested by Kenny (2021). The research revealed that 14 factors had eigenvalues larger than one, with the first component accounting for less than 40% (17.608%) of the variance. As a result, no major common method bias was found in the study (Tables 1, 2).

**Table 1:** Comparative descriptive statistics of the main variables affecting students of both genders.

No.	Construct	α	*M*(SD)	Male *M*(SD)	Female *M*(SD)	*t*
1	PHUB	0.91	2.27(1.01)	2.39(1.01)	2.17(1.01)	3.184^***^
2	FoMO	0.88	3.27(1.32)	3.16(1.30)	3.36(1.33)	−2.192^**^
3	LON	0.96	3.81(1.17)	3.78(1.22)	3.83(1.12)	−2.048^**^
4	SCO	0.95	3.51(1.22)	3.58(1.22)	3.45(1.21)	1.606
5	SMA	0.95	3.8(0.91)	3.79(0.91)	3.8(0.92)	−0.125

SMA, smartphone addiction; PHUB, phubbing behavior; SCO, social comparison orientation; FoMO, fear of missing out. **p < 0.01 and ***p < 0.001.

**Table 2:** Correlation among key variables.

No.	Construct	1	2	3	4	5
1	PHUB	1				
2	FoMO	0.089^**^	1			
3	SCO	0.104^**^	0.116^**^	1		
4	LON	0.187^**^	0.325^***^	0.134^**^	1	
5	SMA	0.240^**^	0.021	0.060	0.129^**^	1

SMA, smartphone addiction; PHUB, phubbing behavior; SCO, social comparison orientation; FoMO, fear of missing out. **p < 0.01 and ***p < 0.001.

### 5.2. Descriptive statistics and correlation analysis

### 5.3. Testing for mediating effect of social comparison orientation

The overall impact of SMA on PHUB and the mediating role of SCO were examined by PROCESS macro (version III) for SPSS. The overall impact of SMA on PHUB is depicted in Figure 1 (total effect = 0.303, *p* = 0.000, 95% CI = [0.2263, 0.3811]). The results show that SMA is positively correlated with SCO (*b* = 0. 236, *p* = 0.000, 95% CI = [0.18, 0.29]), which is in turn positively related to PHUB (*b* = 0.50, *p* = 0.000, 95% CI = [0.38, 0.61]). The direct residual impact was also significant (*b* = 0.19, *p* = 0.000, 95% CI = [0.107, 0.26]). As a result, SCO partially mediated the relationship between SMA and PHUB (indirect effect = 0.118, 95% CI = [0.08, 0.15]). Around 5.7% of the variance in PHUB among Pakistani HE students was explained by this model.

### 5.4. Testing for mediating effect of fear of missing out

The mediating impact of self-control was also investigated *via* PROCESS macro for SPSS. Findings reveal that SMA was positively linked to FoMO (*b* = 0.092, *p* = 0.037, 95% CI = [0.03, 0.15]), which, sequentially, was positively linked to PHUB (*b* = 0.402, *p* = 0.000, 95% CI = [0.26, 0.46]). The residual direct impact was likewise significant (*b* = 0.27, *p* = 0.000, 95% CI = [0.195, 0.34]). Therefore, the effect of SMA on PHUB was partially mediated by FoMO (indirect effect = 0.034, 95% CI = [0.01, 0.06]). This model explained 6.5% of the variance in PHUB among HE students in Pakistan.

### 5.5. Multiple mediation model test

**Figure 2:**
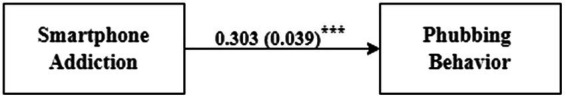
Model of total effect the path coefficients are represented by the path values (standard errors). *** Value of *p* < 0.001.

**Figure 3:**
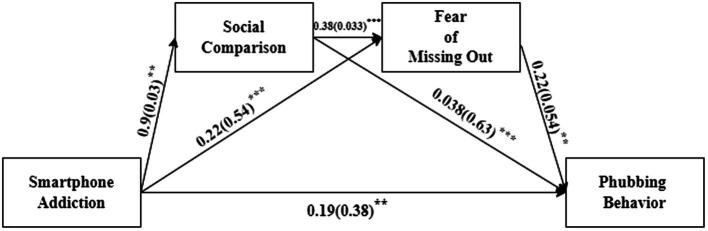
Model of multiple mediations. The path coefficients are the path values (standard errors). ^***^p < 0.001, ^**^p < 0.01.

The multiple mediation model was examined using the PROCESS macro for SPSS (Model 6). Figure 2 depicts the total effect of SMA on PHUBs (total effect = 0.31, *p* = 0.000, 95% CI = [0.236, 0.392]). Table 3; Figure 3 show that all pathways were significant. SCO and FoMO partially mediate the link between SMA and PHUB (SCO: indirect effect = 0.020, 95% CI = [0.004, 0.041]; FoMO: indirect effect = 0.078, 95% CI = [0.0471, 0.112]). The pathway of “SMA/SCO/FoMO/PHUB” was significant (indirect effect = 0.013, 95% CI = [0.0038, 0.0256]), indicating that a higher level of SMA was associated with higher SCO (*b* = 0.201, 95% CI = [0.15, 0.25]), FoMO (*b* = 0.092, 95% CI = [0.03, 0.155]), and a higher tendency to PHUBs. The residual direct impact was likewise significant (*b* = 0.191, 95% CI = [0.12, 0.266]). Consequently, in the effect of SMA on PHUB, SCO, and FoMO only partially performed mediation functions. The multiple mediation model employed in the research contributed significantly to the explanation of the variation in PHUBs among Pakistani HE students (*R*^2^ = 0.23).

**Table 3:** Path analysis of the multiple mediation model.

Path	*B* coefficient	95% CI	
		Lower	Upper
a. Total effect model			
SMA/PHUB	0.3128	0.236	0.392
b. Multiple mediation model			
Direct effects			
SMA/PHUB	0.191	0.115	0.267
SMA/SCO	0.201	0.15	0.25
SCO/PHUB			
SMA/FoMO	0.092	0.03	0.155
FoMO/PHUB	0.22	0.11	0.32
SCO/FoMO	0.38	0.31	0.44
Indirect effects			
SMA/SCO/PHUB	0.020	0.004	0.041
SMA/FoMO/PHUB	0.078	0.0471	0.112

SMA, smartphone addiction; PHUB, phubbing behavior; SCO, social comparison orientation; FoMO, fear of missing out. *N* = 884.

### 5.6. Moderated mediation model test

The current study anticipated that, through SCO and FoMO, loneliness would mitigate the direct and secondary impacts of SMA on PHUB. To investigate the moderated mediation hypothesis, the PROCESS macro v.3 for SPSS was utilized (Model 8). The findings are shown in Table 4. The findings indicate that the interaction between SMA and loneliness on SOC (*b* = 0.038, 95% CI = [−0.012, 0.0889], *p* = 0.137) and on FoMO (*b* = 0.046, 95% CI = [−0.0028, 0.096], *p* = 0.064) are insignificant. This suggests that loneliness does not influence the direct and indirect effects of SMA on FoMO and SOC.

**Table 4:** Moderated mediation analyses’ results (PROCESS: model 8).

Model 1: Mediator variable model	Outcome: SCO				Bootstrapped CI (95%)	
	Coeff	SE	*t*	*p*	LL	UL
SMA	0.295	0.0421	7.003	0.000	0.212	0.377
LON	0.325	0.042	10.28	0.000	0.263	0.387
SMA × SC	0.038	0.025	1.488	0.137	−0.012	0.0889
Model 2: Mediator variable model	Outcome: FoMO					
					Bootstrapped CI (95%)	
	Coeff	SE	*t*	*p*	LL	UL
SMA	0.24	0.41	5.83	0.000	0.159	0.321
LON	0.202	0.031	6.52	0.000	0.141	0.262
SMA × LON	0.046	0.025	1.85	0.064	−0.0028	0.096
Model 3: Outcome variable model	Outcome variable: PHUB					
SMA	0.314	0.033	9.52	0.000	0.249	0.379
SCO	0.187	0.038	4.94	0.000	0.113	0.262
FoMO	0.044	0.038	1.14	0.254	−0.032	0.120
LON	0.125	0.025	4.87	0.000	0.075	0.176
SMA × LON	−0.0858	0.0193	−4.45	0.000	−0.123	−0.047
Conditional indirect effect (*via* SCO and FoMO)					Bootstrapped CI (95%)	
	Coeff	SE	*t*	*p*	LL	UL
LON(+1 SD)	0.450	0.044	10.13	0.000	0.363	0.537
LON (−1 SD)	0.177	0.0455	3.906	0.0001	0.088	0.267
	Index	SE			LL	UL
Index of moderated mediation	−0.02	0.01			−0.05	−0.001

SMA, smartphone addiction; PHUB, phubbing behavior; SCO, social comparison orientation; FoMO, fear of missing out; LON, loneliness.

**Figure 4:**
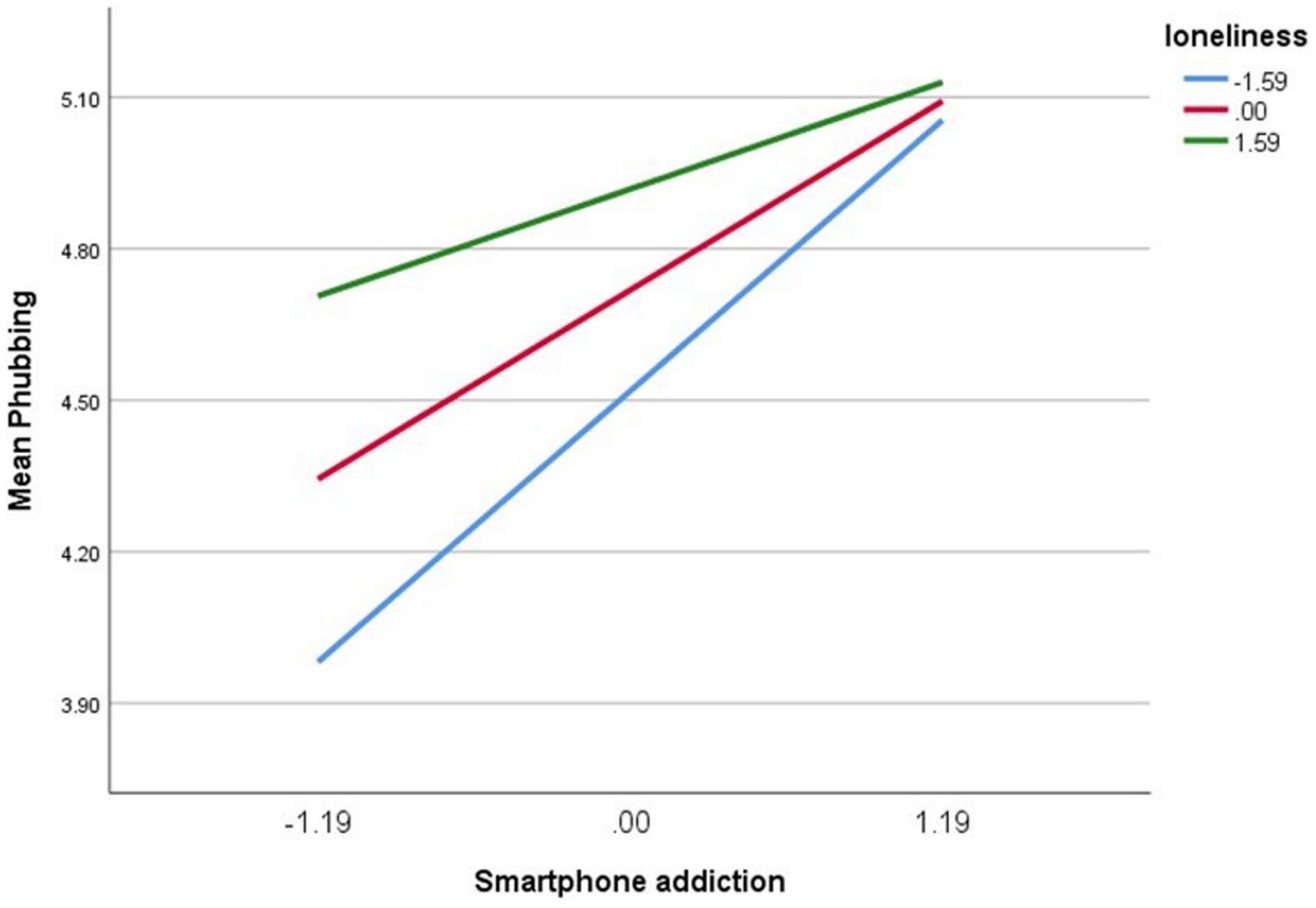
The moderating impact of loneliness on the relationship between SMA and PHB.

The findings of the moderated mediation reveal that loneliness would moderate the indirect associations between SMA and PHUB, as shown in Table 4 (index = −0.02, boot SE = 0.01, 90% CI = [−0.05, −0.001]). Figure 4 displays further findings from a simple slope analysis. SMA has a substantial positive predictive influence on PHUB. However, this effect was less pronounced for participants with low levels of loneliness (M – 1 SD) (*b*_simple_ = 0.177, *t* = 3.906, *p* < 0.001). For participants with high loneliness levels (M + 1SD), job burnout also has a positive predictive effect on depression (*b*_simple_ = 0.450, *t* = 10.13, *p* < 0.000), indicating that with the improvement of loneliness level, the predictive effect of SMA on PHUB gradually increases.

## 6. Discussion

The current study focused on Pakistani university students and sought to investigate the moderated mediation roles of SCO, FoMO, and loneliness in the association between SMA and PHUB. Although the relationship between FoMO, SCO use, and/or SMA has been studied, this is the first known study that investigates the relationships among all these variables and probes into the mediating or moderating mechanisms. The findings indicated that SMA was positively associated with PHUB. Furthermore, FoMO and SCO sequentially mediated the relationship between PHUB and SMA, and that loneliness might act as a moderator between PHUB and SMA, implying that this relationship was stronger for university students with high loneliness than for those with low loneliness. Finally, the results revealed that SMA affected PHUB *via* FoMO and SCO with the increment of loneliness.

As expected, SMA’s possible involvement as a contributor to PHUB dysfunction is corroborated by the fact that it has a positive relationship with PHUB. Furthermore, high scores on SMA are associated with higher scores of PHUBs, which is in line with prior studies (Franchina et al., 2018; Błachnio and Przepiorka, 2019; Nazir and Bulut, 2019). One issue that appears in the digital age and needs many people’s attention, including counselors and other education professionals, is PHUB. Therefore, complementary programs are needed to educate HE students on how to use their smartphones wisely. One possible idea is to develop awareness campaigns consisting of two-day workshops and lectures to comprehend the occurrence of PHUB and equip therapists with possible remedial tools. Through these awareness sessions, consular officials may acknowledge SMA and PHUB as real problems faced by students and raise awareness of the dangers of smartphone abuse among them.

Another possible remedy is to offer courses where students who are hooked to smartphones (and those who do not) may receive counseling services on campus. One of the best ways to prevent excessive smartphone usage is to give up the device for 1 day and engage in rejuvenating activities. This may help users cut down on their need for the device, foster offline social connections, and nurture relationships with new acquaintances (Zaremohzzabieh et al., 2015). This study also expects that PHUB may be overcome through the implementation of counseling services in the learning process (Afdal et al., 2018). These proposed remedies can therefore be used as the ideal solution to the entire issue, encouraging students to be more productive and healthier while using smartphones, to have a greater understanding of how real and virtual worlds interact, to exercise self-control while using smartphones, to further develop their sense of social responsibility, and to spread awareness of SMA.

Meanwhile, the results from the research on the mediation impacts of SCO and FoMO are consistent with previous research (Reer et al., 2019). According to the findings, the total effect of SMA on PHUB is distributed across both the direct and indirect association mechanisms of SCO and FoMO. This finding adds to our understanding of the mechanisms or pathways that lead to PHUB among HE students. HE students will be concerned about losing the emotional support that they receive from their smartphones if they leave them for an extended period, effectively developing a FoMO. The Pakistani population has a collectivistic culture with a lifestyle that emphasizes increased socialization and interdependence. This means that Pakistani people tend to have large social networks with whom they feel strongly affiliated and obligated to keep in touch (Curnutt, 2007). They see themselves as a part of their social circles – missing any event or conversation can make them feel as though they are missing out (Butt and Arshad, 2021). Additionally, according to Butt and Arshad (2021), a smartphone can be viewed as a status symbol due to SC orientation, a tool of connection due to extended social groups, and an essential component of life for people living in Pakistan to maintain virtual connections with their social group.

As a result, FoMO may increase HE students’ insecurity and encourage them to overuse their smartphones to alleviate negative moods, eventually leading to PHUB (Franchina et al., 2018). This finding also indicates that HE students with high levels of SCO are more likely to engage in more intense SC behaviors. This would in turn increase their fear of being left behind in social contexts. Moreover, the risk of an escalating relationship between SMA and PHUB is even more likely. It should be noted that such mediation was found to be partial given that the direct path from HE students’ predispositions to SMA and PHUB linkage remains significant.

It is important to note that lecturers frequently utilize SM platforms to advertise role models at work, encourage students to socially compare themselves to exceptional students, and promote learning from them. The current research suggests that lecturers should exercise caution when adopting this approach since doing so might lead students to experience ego depletion and unanticipated performance problems. By exercising more caution, teachers may sensibly encourage students to prevent peer pressure brought on by SC. To properly manage this material with a more at ease mind, lecturers must also have a right view of the significance of shared information on smartphones and frequently remind themselves that the content posted on social platforms should be beneficial and good.

Finally, the findings imply that loneliness moderates the indirect relationships between SMA and PHUB. The authors of this study believe that these positive moderation effects may be attributed to the fact that HE students – who experience higher levels of loneliness while using smartphones – may be anxious about missing potential likes or social rewards for their posts in comparison with others (Rosenthal-von der Pütten et al., 2019). Subsequently, these students tend to engage in SCO and FoMO-driven internet usage to avoid being alone. In addition, HE students who feel lonely will have an increased likelihood of PHUB, as the world of smartphones makes them feel connected to other students (Savci and Aysan, 2017). In reality, the PHU phenomenon does not help HE students to alleviate their loneliness. It increases their levels of loneliness through a reduced sense of community (Ang et al., 2019). Awareness-raising training may be given to parents and educators to reduce HE students’ feelings of loneliness. In this way, they may better understand the typical conflicts faced by young adults similar to them. These measures, which may reduce students’ feelings of loneliness, will also contribute to the reduction of their SMA and PHUB.

## 7. Conclusion

Given the rising concern about SMA and how it impacts HE students in Pakistan, this research adds considerably to the body of knowledge on its negative consequences. The goal of the current study is to better understand how SMA affects PHUB. Additionally, this study provided proof for the moderating influence of loneliness as well as evidence for the partial mediation effects of SCO and FoMO. Therefore, the study’s conclusions have significant consequences for both theory and practice.

## 8. Limitations and direction for future studies

Several significant limitations of the present study must be recognized, despite the findings providing encouraging empirical evidence for our integrative model incorporating SMA, PHUB, SCO, FoMO, and loneliness. Firstly, a proper cause-and-effect conclusion cannot be established, as the current study is cross-sectional. This is because psychological symptoms can contribute to loneliness, PHUB, FoMO, and SCO. Secondly, it would be beneficial to assess this model’s applicability in a clinical sample. The degree of the additive and interaction effects of FoMO, SCO, and loneliness on PHUB in a clinical setting may be considerably different from what was observed in the current sample. This is although we would typically have the same or comparable set of expectations for assessing PHUB in a clinical sample.

Researchers may also examine how sociodemographic characteristics may have an impact on PHUB. Fourthly, the current study relies on self-reported data from a particular nation (Pakistan), limiting its findings’ generalizability. To get more generalizable insights into the correlations being studied, the current study’s approach might be reproduced using samples from varied sociodemographic groups and nations. Last but not least, the study used a cross-sectional methodology, which leaves the findings open to potential biases. Future research may focus on longitudinal and observational research to eliminate such biases and to examine how these factors interact while considering temporal effects.

## Data availability statement

The raw data supporting the conclusions of this article will be made available by the authors, without undue reservation.

## Ethics statement

Ethical review and approval was not required for the study on human participants in accordance with the local legislation and institutional requirements. The patients/participants provided their written informed consent to participate in this study.

## Author contributions

RS: data collection. HA and ZZ: supervision and draft. ZZ: data analysis and review. AA and WW: visualization. All authors contributed to the article and approved the submitted version.

## Conflict of interest

The authors declare that the research was conducted in the absence of any commercial or financial relationships that could be construed as a potential conflict of interest.

## Publisher’s note

All claims expressed in this article are solely those of the authors and do not necessarily represent those of their affiliated organizations, or those of the publisher, the editors and the reviewers. Any product that may be evaluated in this article, or claim that may be made by its manufacturer, is not guaranteed or endorsed by the publisher.
